# rPIMS: a ShinyR package for the precision identification and modelling of livestock breeds using genomic data and machine learning approaches

**DOI:** 10.1093/bioadv/vbaf077

**Published:** 2025-04-07

**Authors:** Yuhetian Zhao, Xuexue Liu, Benmeng Liang, Lin Jiang

**Affiliations:** State Key Laboratory of Animal Biotech Breeding, Institute of Animal Sciences, Chinese Academy of Agricultural Sciences (CAAS), Beijing 100193, People’s Republic of China; Centre d’Anthropobiologie et de Génomique de Toulouse, CNRS UMR5288, Université Paul Sabatier, Toulouse 31000, France; Institute of Molecular Medicine, Renji Hospital, School of Medicine, Shanghai Jiao Tong University, Shanghai 200127, People’s Republic of China; State Key Laboratory of Animal Biotech Breeding, Institute of Animal Sciences, Chinese Academy of Agricultural Sciences (CAAS), Beijing 100193, People’s Republic of China

## Abstract

**Summary:**

Accurate breed identification serves is a crucial cornerstone for the conservation and utilization of livestock and poultry genetic resources. The identification of breeds based on a variety of information sources and analytical methods has been extensively applied in the domain of animal genetics and breeding. Recently, the integration of large-scale genomic data with machine learning has become increasingly prevalent for breed identification tasks. However, such projects typically require extensive sequencing data and expertise in bioinformatics. To address this, we introduce rPIMS, a comprehensive tool designed to simplify breed identification and genetic analysis. With intuitive modules for data input, dimensionality reduction, phylogenetic tree construction, population structure analysis, and machine learning-based classification, rPIMS has the capacity to streamlines the analytical process for researchers. It promotes collaboration, facilitates efficient data sharing, and enhances the ability to identify and report genetic diversity and evolutionary relationships among livestock breeds. We performed a validation analysis to confirm that rPIMS achieved 100% classification accuracy in distinguishing 10 breeds using only 860 SNPs. In summary, rPIMS significantly simplifies complex model-building processes, making breed classification and genetic structure visualization accessible and intuitive to users.

**Availability and implementation:**

rPIMS is a Shiny R application designed for breed identification in livestock using genomic data and machine learning, accessible through an intuitive graphical user interface. It is freely available under the GNU Public License on GitHub: https://github.com/Werewolfzy/rPIMS.

## 1 Introduction

Livestock and poultry resources represent a vital component of global genetic diversity, with local breeds providing distinctive traits and serving as key reservoirs of valuable genetic material (Williams *et al.* 2016, [Bibr vbaf077-B39][Bibr vbaf077-B39]). These breeds often possess unique adaptations to their specific environments, making them an essential resource for maintaining biodiversity and resilience in the face of changing conditions (Zhao *et al.* 2021, Restoux *et al.* 2022). However, many of these local breeds, which contribute significantly to the genetic diversity of livestock populations, are increasingly threatened by factors such as disease outbreaks, extreme weather conditions, and human intervention (Yaro *et al.* 2017, Miao *et al.* 2023). In addition, current limitations in breed identification technologies have resulted in market confusion, where inferior breeds are mistakenly marketed or perceived as superior, negatively affecting breed quality and economic outcomes (Liang *et al.* 2023). In this context, the precise identification of these breeds plays a pivotal role in their conservation and sustainable management, enabling effective strategies to protect and utilize their genetic potential in breeding programs and agricultural practices.

Genetic diversity in livestock is primarily due to genetic differences, mainly in the form of single nucleotide polymorphisms (SNPs). Because of their widespread distribution and high density throughout the genome, SNP markers have been widely used for analyses of genetic structure (Berihulay *et al.* 2019, Islam *et al.* 2019, Oget *et al.* 2019, Wang *et al.* 2022, Nantongo *et al.* 2024), identification of genes associated with economically important traits (Raza *et al.* 2020, Li *et al.* 2024, Niu *et al.* 2024), and priority conservation assessments (Gao *et al.* 2023a,b, Zhang *et al.* 2023, Arias *et al.* 2024). Furthermore, the employment of SNPs in the identification of livestock breeds is becoming increasingly widespread. The employment of high-throughput SNP chips or whole-genome sequencing technologies facilitates genome-wide screening and genotyping of livestock populations (Zhao *et al.* 2023a). Initially, there were attempts to combine SNP markers with PCR restriction fragment length polymorphism and minor allele frequency linkage disequilibrium in different livestock for generating panels for target breed identification (Suekawa *et al.* 2010, Ramos *et al.* 2011, Brooks *et al.* 2016, Zhao *et al.* 2024). However, with the advent of artificial intelligence and machine learning, new possibilities have emerged for the utilization of genomics information and various classification models to identify diverse animal populations. Research in this area has been undertaken for pigs (Pasupa *et al.* 2020, Schiavo *et al.* 2020, Gao *et al.* 2022, Miao *et al.* 2023), chickens (Seo *et al.* 2021, Cho *et al.* 2022), and cattle (Bertolini *et al.* 2018, Zhao *et al.* 2023a) by using machine learning. However, previous studies have mostly focused on single species or commercial multi-breed populations, often requiring large training datasets. This approach is not well-suited for local breeds with smaller population sizes, where existing models cannot be readily applied. rPIMS offers the first interactive platform for training multi-breed classification models from any species, making it more accessible for such cases. It not only helps users identify core breeds based on genetic structure but also enables the use of a minimal number of loci to build models, improving prediction accuracy and reducing processing time. Additionally, it enhances the collaborative experience by allowing all stakeholders and project partners to directly interact with the data, fostering better communication and cooperation.

## 2 Implementation

**Figure 1.**
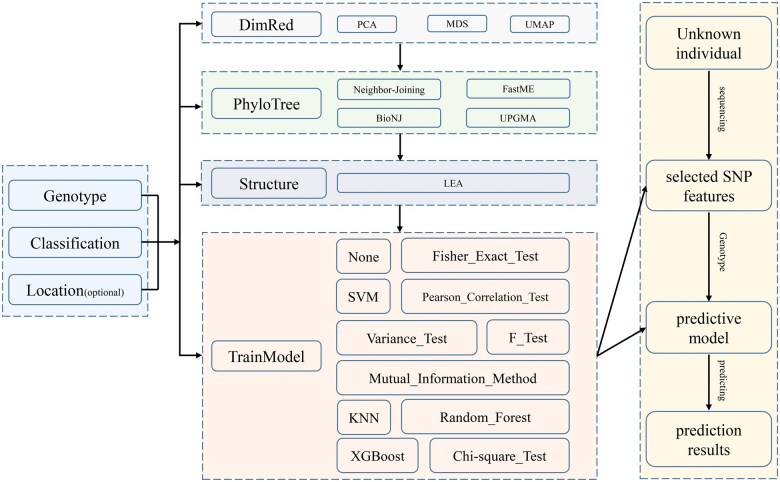
Workflow of the rPIMS packages.

The rPIMS framework is specifically engineered to investigate and elucidate breed-specific genomic patterns through the integration of advanced machine learning techniques with comprehensive visualization tools. This R-package is designed to develop accurate classification and predictive models for various breeds while optimizing cost-efficiency, primarily through the strategic reduction of featured SNPs. A detailed workflow diagram illustrating this process is presented in Fig. 1. The software accepts three input files: genomic data in Hapmap format, breed information with columns labelled ‘ID’ and ‘breed’ and an optional geographic data file with columns labelled ‘breed’, ‘Latitude’, ‘Longitude’, and ‘Location’. Through its modular interface, users can interactively implement the following functions:

- Data Visualization and Pre-analysis Check (DATA): This module allows users to visualize imported genomic data, breed-related information, and geographic sampling data. It performs an initial format validation of input files and provides statistical power analyses to recommend a minimum number of individuals per breed. For breeds with insufficient sample sizes, the module offers optional upsampling or downsampling methods to balance individual counts across breeds prior to subsequent analyses.
- Dimensionality Reduction Analysis (DimRed): This module provides three dimensionality reduction methods: Principal Component Analysis (PCA) (Pearson 1901), Multidimensional Scaling (MDS) (Dixon 2003), and Uniform Manifold Approximation and Projection (UMAP) (McInnes *et al.* 2018), for exploring genetic relationships among breeds. Each individual is represented by a distinct point colored according to user-defined breed groupings. The resulting visualizations clearly depict breed differentiation or overlap within the reduced-dimensional space. Users can iteratively remove outliers to refine and instantly regenerate PCA, MDS, or UMAP plots without re-uploading the dataset.
- Phylogenetic Tree Analysis (PhyloTree): This module uses four distinct methods to construct and visualize phylogenetic trees (Paradis and Schliep 2019): Neighbor-Joining, FastME, BioNJ, and UPGMA. This module enables users to explore evolutionary relationships among breeds. Each tree can be customized by selecting color schemes to distinguish breeds, with node support values clearly indicating the reliability of branch structures. Users can iteratively remove specific outlier individuals to update the phylogenetic tree without re-uploading the dataset.
- Population Structure Analysis (Structure): This module implements population structure analysis to infer genetic ancestry and diversity within and across breeds. Users can explore ancestral components by determining the optimal number of genetic clusters (*K*) through cross-validation. Results are visualized clearly, presenting individual-level admixture proportions with customizable color schemes, facilitating intuitive interpretation of genetic differentiation and ancestry among breeds.
- Feature Selection and Model Training (TrainModel): This module enables the construction of predictive breed classification models by integrating feature selection and machine learning techniques. Multiple feature selection methods—including mutual information, correlation-based filtering, and statistical tests such as Fisher’s exact test, Chi-square test, mutual information, and entropy-based measures—are provided to reduce redundant SNPs. Various machine learning algorithms, including K-Nearest Neighbors (KNN) (Venables and Ripley 2013), Random Forest (RF) (Liaw and Wiener 2002), Support Vector Machine (SVM) (Meyer *et al.* 2023), and Extreme Gradient Boosting (XGBoost) (Chen *et al.* 2024), are available for model training. To enhance generalization and reliability, several cross-validation methods are incorporated, such as k-fold, randomized k-fold, and leave-one-out cross-validation. Users can flexibly combine these feature selection methods and classification models to achieve optimal predictive performance. Additionally, users have the option to manually reduce SNPs to obtain a more precise and smaller set of SNPs.
- Breed Prediction for New Individuals (PredNewind): This module applies pre-trained classification models to genomic data from unknown individuals to accurately predict breed assignment. An integrated one-class validation procedure ensures robust predictions by effectively excluding random or low-quality genotypes. Results include not only breed assignments but also calibrated accuracy scores, enabling users to evaluate prediction reliability. Additionally, geographic distributions of predicted breeds are visualized clearly, providing insights into spatial patterns of genetic diversity.

Users can customize parameters such as color schemes, number of ancestral components in structure analysis and selection of breeds for model training. The download feature allows facilitates the generation of comprehensive visualizations and reports in multiple formats (e.g. PDF, PNG, TIFF) based on user-defined analysis settings. rPIMS provides flexibility by allowing users to refine their dataset in steps 2, 3, and 4 by removing outliers within a breed to improve prediction accuracy. Alternatively, users can skip these steps and proceed directly to the core function in step 5—model training.

In the field of machine learning, the inclusion of additional feature SNPs does not always lead to better results. The introduction of irrelevant features has been demonstrated to result in a degradation of model performance (Li *et al.* 2023). Consequently, rPIMS incorporates various biostatistician methods to first identify correlations between loci and eliminate redundant data, resulting in a refined set of featured loci. Subsequently, machine learning algorithms further reduce loci with low contributions to the model, ultimately producing an accurate prediction model with a concise set of SNP markers. These breed-specific SNP marker sets can also assist in the development of breeding chips, enhancing their application in genetic improvement programs.

## 3 Application

### 3.1 Data source

To demonstrate the flexibility of rPIMS, we have provided three example files that illustrate the analysis capabilities of this package. These data are derived from the study conducted by Liang *et al.* (2023) and are based on Illumina Ovine SNP50 chip data. To simplify usage, we randomly selected 10 breeds. The data underwent basic cleaning using Plink (Purcell *et al.* 2007), where loci with a high missing rate were filtered out using the parameters—geno 0.1, maf 0.01, mind 0.1, and hwe 0.00001. Additionally, linkage disequilibrium pruning was performed using the parameter—indep 50 5 2. The first file contains genomic SNP data for 177 individuals across the 10 breeds (genotype.hmp.txt, Supplementary Document Fig. S1), with 145 486 SNPs, which was converted from a genomic VCF file using the TASSEL5 software (Bradbury *et al.* 2007). The tabular format of the data facilitates R processing and analysis. The second file (classification.txt, Supplementary Document Fig. S2) provides detailed breed classifications, linking each of the 177 individuals to a specific breed. The third file (location.txt, Supplementary Document Fig. S3) includes geographic coordinates (latitude and longitude) of sampling locations for the 10 breeds, adding spatial context to the analysis. Statistical power analysis confirmed that the sample sizes for all breeds exceeded the recommended thresholds, allowing the analysis to proceed to the next step without adjustments (Supplementary Table S1, Supplementary Document Fig. S4).

### 3.2 Population structure visualization

Dimensionality reduction analysis was performed on the input dataset. The PCA (PC1 and PC2, Supplementary Fig. S1) collectively accounted for 7.13% of total genetic variance, with all individuals residing within breed-specific confidence intervals, demonstrating the absence of significant outliers requiring curation. The PCA clustering revealed pronounced genetic differentiation among breeds (Supplementary Fig. S1, Supplementary Document Fig. S6).

The result of the phylogenetic tree (Supplementary Fig. S2, Supplementary Document Fig. S7) provides further evidence in support of these findings, with individuals from the same breed forming discrete and well-separated clusters. The breed-specific clustering pattern suggests strong genetic cohesion within breeds and clear differentiation between them, thereby reinforcing the reliability of the classification approach.

As demonstrated in Supplementary Fig. S3A shows the cross-validation error for varying K values is depicted, thereby identifying *K* = 7 as the optimal number of ancestral components based on the lowest error. Supplementary Fig. S3B illustrates the admixture proportions of each individual at *K* = 7, providing insights into genetic composition and admixture levels among breeds. The results indicate that while some breeds exhibit high genetic homogeneity, others show varying degrees of admixture, reflecting potential historical gene flow. These observations are consistent with the PCA results. For an overview of the analytical workflow, refer to Supplementary Document Fig. S8.

### 3.3 Feature selection and model construction

Subsequent to conducting population genetic structure analysis, we performed feature selection and model construction using the software’s default parameters (Supplementary Document Fig. S9). In the given example, approximately 5000 SNPs were initially obtained using the automated procedure. After manual selection and adjustment, the number of SNPs was reduced to the top 17% (860 SNPs), which still achieved optimal prediction results (AUC = 1, Accuracy = 1, Kappa = 1), as illustrated in Supplementary Fig. S4. It should be noted that, if necessary, users have the option to further reduce the number of SNPs in order to minimize the cost and effort associated with sequencing, but with the understanding that this may result in a slight compromise in accuracy. The selected SNPs can also be used in the development of breeding chips, increasing their applicability in livestock breeding.

To further evaluate predictive power of the model, we tested it using genomic data from 10 unknown breed individuals. The results (Supplementary Table S2, Supplementary Document Fig. S10) showed that all individuals successfully passed one-class validation and obtained high z-score values, indicating high prediction confidence and model robustness. The z_score represents the calibrated accuracy of the breed prediction, with higher z_scores indicating higher prediction accuracy. It is calculated as follows:

(1) $$z\_\mathrm{score}=\frac{P^{\alpha }}{P^{\alpha }+{\left(1-P\right)}^{\alpha }}+\alpha \;•\;(0.5-\frac{P^{\alpha }}{P^{\alpha }+{\left(1-P\right)}^{\alpha }}),\;\text{if}\;P\leq 0.5$$

 

(2) $$z\_\mathrm{score}=\frac{P^{\alpha }}{P^{\alpha }+{\left(1-P\right)}^{\alpha }},\;\text{if}\;P>0.5$$

where *P* is the original predicted probability, and α is a tuning parameter. This formula adjusts the original predicted probabilities, improving the calibration and providing a more balanced representation of the prediction accuracy.

Detailed installation instructions, input file format guides, interface examples, and descriptions of additional features for rPIMS can be found in the supplementary materials.

## 4 Conclusion

The rPIMS enables interactive analysis of livestock genomic data, offering an intuitive platform for exploring genetic diversity and breed relationships. It provides researchers with a comprehensive toolset to visualize genetic and geographic patterns across breeds, facilitating the identification of breed-specific markers and informing conservation strategies. Additionally, rPIMS enhances collaborative efforts by making complex genomic analyses accessible to users without advanced bioinformatics expertise, allowing diverse stakeholders to actively engage in data exploration and breed identification efforts.

## Supplementary Material

vbaf077_Supplementary_Data

## Data Availability

The data underlying this article are available in *GitHub* at https://github.com/Werewolfzy/rPIMS.
