# Supplementary material for: rPIMS: a ShinyR package for the precision identification and modelling of livestock breeds using genomic data and machine learning approaches
Source: Bioinform Adv. 2025 Apr 7;5(1):vbaf077. doi: 10.1093/bioadv/vbaf077 (PMC12052404; doi:10.1093/bioadv/vbaf077)
Supplement: vbaf077_Supplementary_Data [file vbaf077_supplementary_data.zip › supplementary_data.docx]

**Table S**

**Table S1.** The results were obtained through statistical power analysis performed using the DATA module of the rPIMS software.

| Group | Current_Sample | Recommended | Need_Supplement |
| --- | --- | --- | --- |
| DormaSheep | 19 | 9 | No |
| Gangbasheep | 19 | 9 | No |
| Gonggasheep | 18 | 9 | No |
| Huoerbasheep | 19 | 9 | No |
| Heizangsheep | 13 | 9 | No |
| Jianchuansheep | 18 | 9 | No |
| Jiangzisheep | 18 | 9 | No |
| Ninglangsheep | 18 | 9 | No |
| Shangusheep | 18 | 9 | No |
| Zuogongsheep | 17 | 9 | No |

**Table S2.** The results obtained from predictions using the PredNewind module of the rPIMS software.

| IID | predicted_result | z_score | confidence | Latitude | Longitude | Location |
| --- | --- | --- | --- | --- | --- | --- |
| Gonggasheep1 | Gonggasheep | 0.99 | Reliable | 29.9124 | 102.2332 | Luding County, Garze, Sichuan |
| Gonggasheep2 | Gonggasheep | 1.00 | Reliable | 29.9124 | 102.2332 | Luding County, Garze, Sichuan |
| Heizangsheep1 | Heizangsheep | 1.00 | Reliable | 35.5885 | 100.7498 | Guinan County, Hainan, Qinghai |
| Heizangsheep2 | Heizangsheep | 1.00 | Reliable | 35.5885 | 100.7498 | Guinan County, Hainan, Qinghai |
| Huoerbasheep1 | Huoerbasheep | 0.80 | Reliable | 29.7699 | 84.1589 | Zhongba County, Shigatse, Tibet |
| Huoerbasheep2 | Huoerbasheep | 0.59 | Reliable | 29.7699 | 84.1589 | Zhongba County, Shigatse, Tibet |
| Jianchuansheep1 | Jianchuansheep | 0.75 | Reliable | 26.5457 | 99.905 | Jianchuan County, Dali, Yunnan |
| Jianchuansheep2 | Jianchuansheep | 0.75 | Reliable | 26.5457 | 99.905 | Jianchuan County, Dali, Yunnan |
| Jiangzisheep1 | Jiangzisheep | 0.95 | Reliable | 28.91 | 89.6055 | Jiangzi County, Shigatse, Tibet |
| Jiangzisheep2 | Jiangzisheep | 0.99 | Reliable | 28.91 | 89.6055 | Jiangzi County, Shigatse, Tibet |

**Figure S**


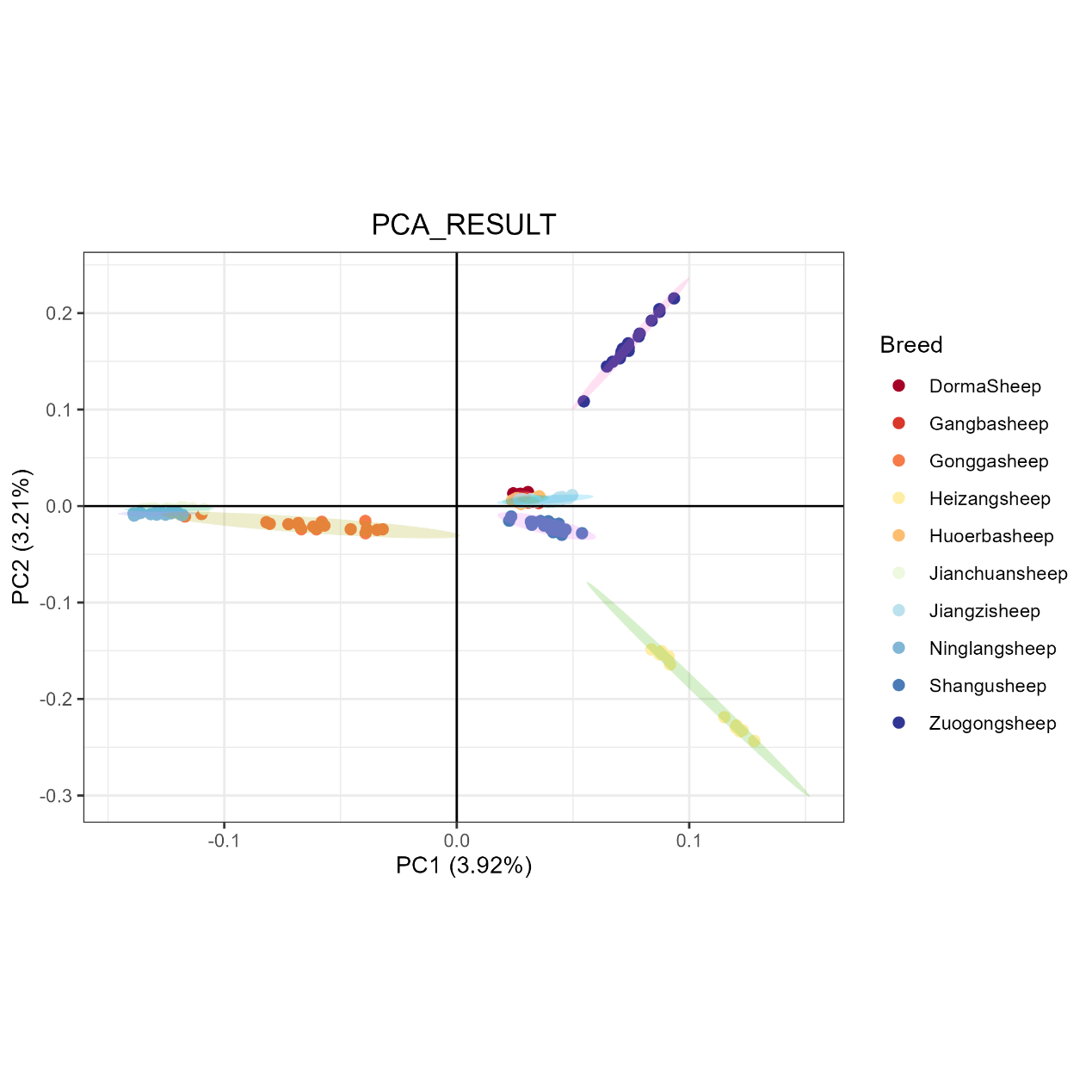


**Figure S1** The PCA dimensionality reduction results obtained using the DimRed module of the rPIMS software.


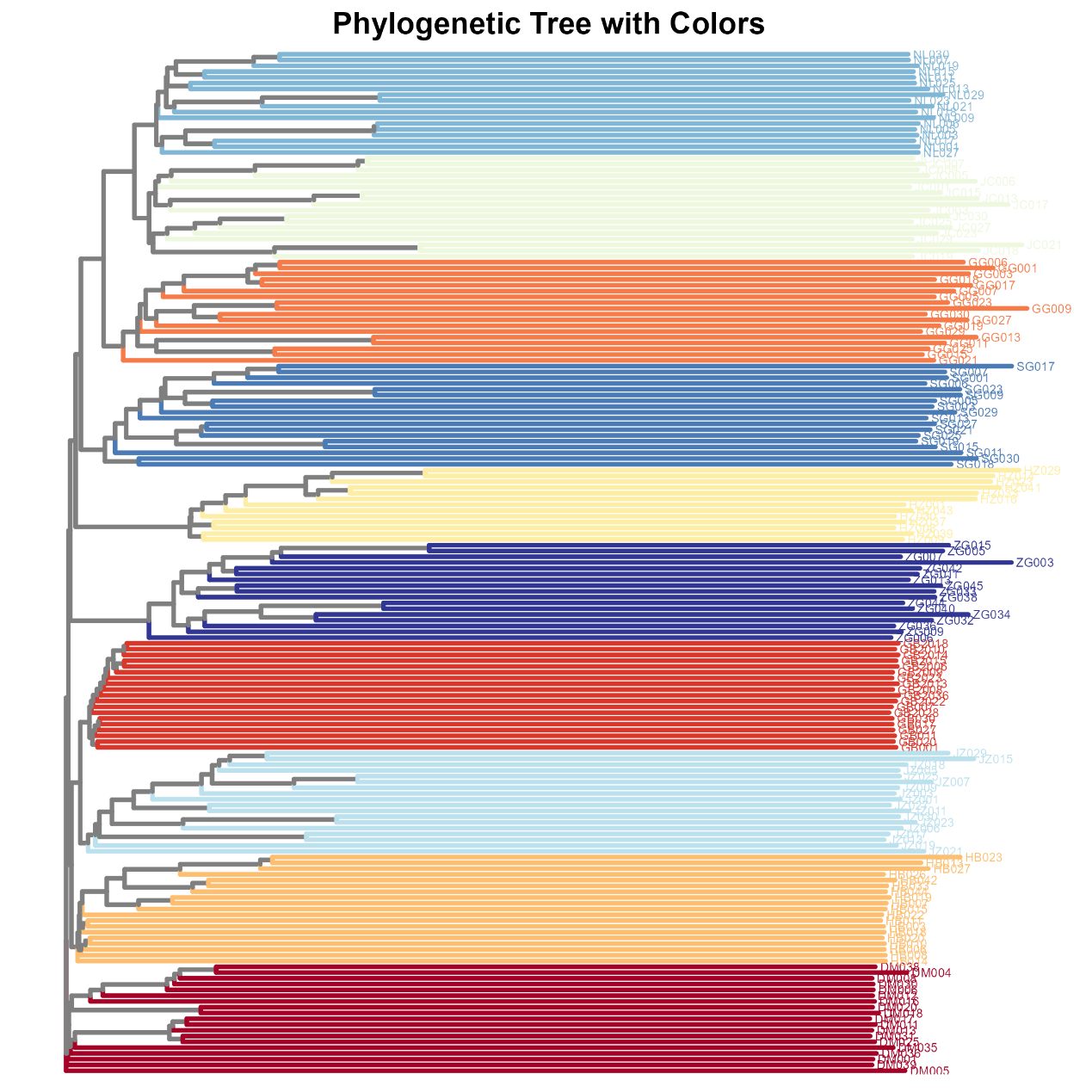


**Figure S2** The population phylogenetic tree results obtained using the PhyloTree module of the rPIMS software.


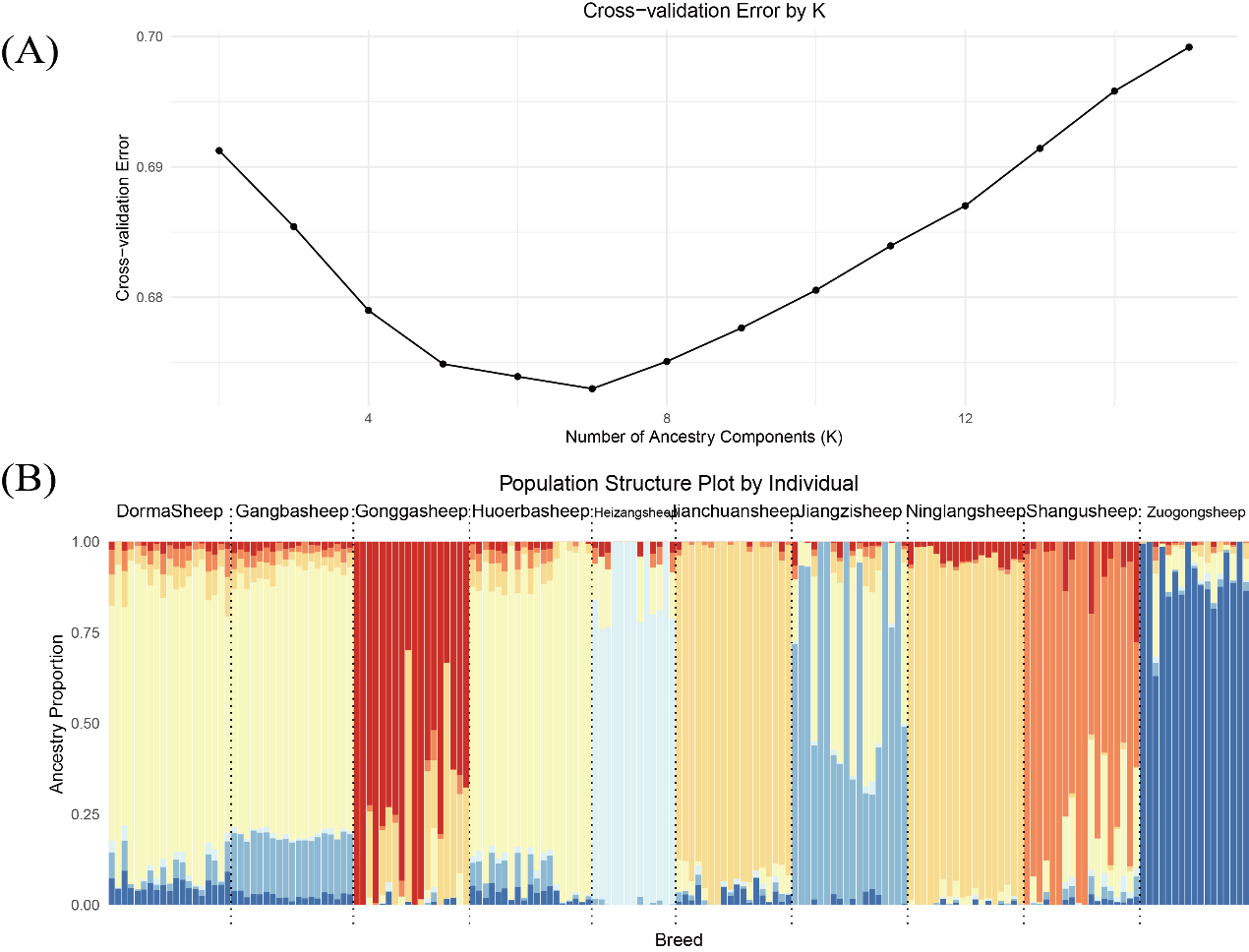


**Figure S3** The population structure analysis results obtained using the Structure module of the rPIMS software. (A) The cross-validation error plot shows the error rates for different K values. (B) Under the optimal K value, the population structure plot displays the proportion of each individual's ancestral components.


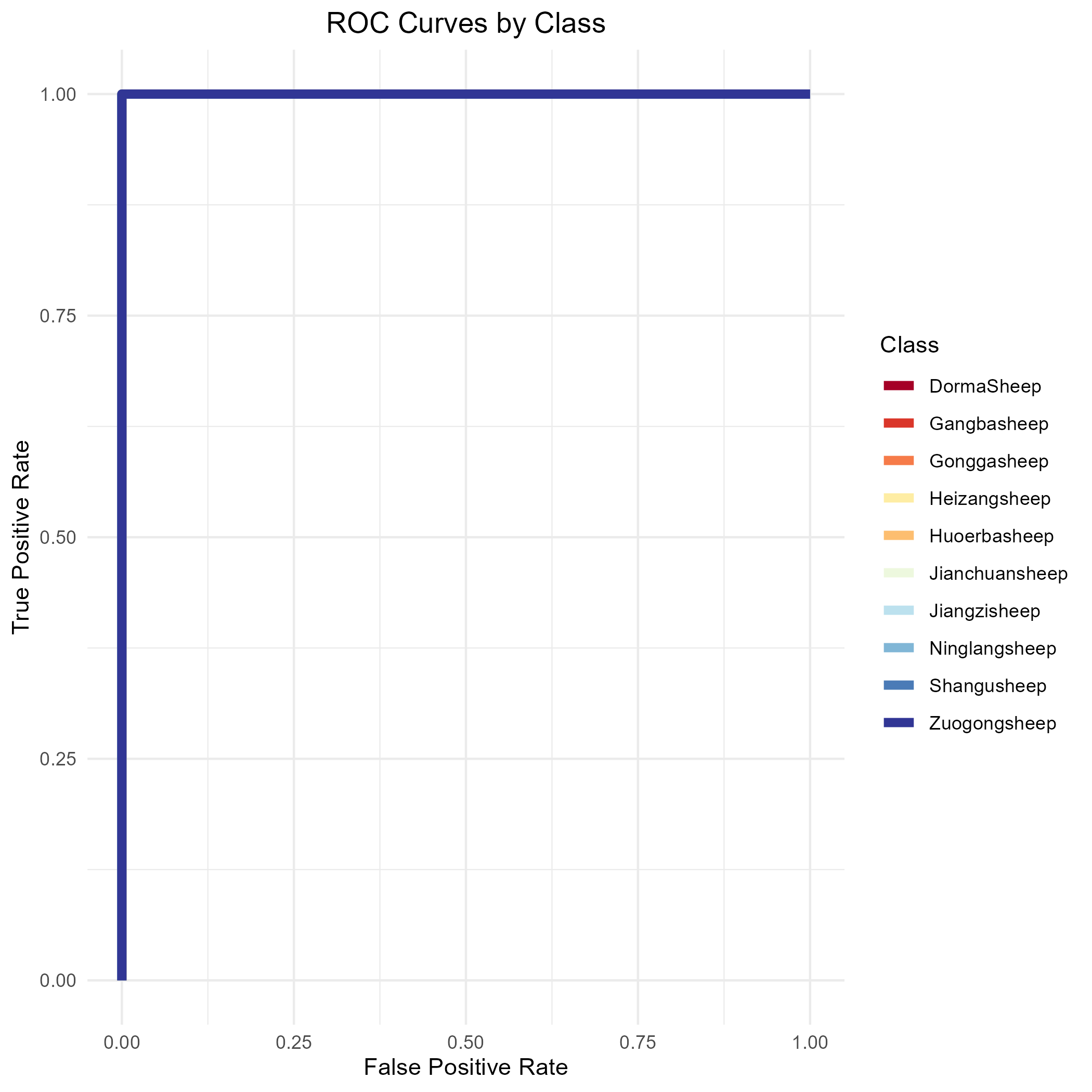


**Figure S4** The ROC curve of the model trained using the TrainModel module of the rPIMS software (the colors overlap because all 10 breeds achieved an AUC of 1).
