# Supplementary material for: rPIMS: a ShinyR package for the precision identification and modelling of livestock breeds using genomic data and machine learning approaches
Source: Bioinform Adv. 2025 Apr 7;5(1):vbaf077. doi: 10.1093/bioadv/vbaf077 (PMC12052404; doi:10.1093/bioadv/vbaf077)
Supplement: vbaf077_Supplementary_Data [file vbaf077_supplementary_data.zip › supplementary_materials.pdf]

rPIMS instructions

Table of Contents

About rPIMS....2

Installing R and R dependencies....2

Running rPIMS....3

Description of Example Files....3

General Instructions....4

- PANEL1: DATA....4
- PANEL2: DimRed....10
- PANEL3: PhylpTree....12
- PANEL4: Structure....14
- PANEL5: TrainModel....16
- PANEL6: PredNewind....20

About rPIMS

The genetic diversity of livestock breeds represents a significant genetic resource. Nevertheless, a number of these breeds are currently at risk of extinction due to a number of challenges, including the emergence of new diseases, climate change and competition from international commercial breeds. It is imperative that accurate breed identification be conducted in order to implement effective measures to mitigate this trend.

The rPIMS package has been developed with the objective of providing breed conservationists with a comprehensive and convenient process for rapidly and accurately constructing breed identification models using a multitude of machine learning techniques. The package is designed to accept both genomic data and simple tabular text files as input, integrating a diverse range of analysis modules to facilitate comprehensive and precise breed identification.

Including:

(1) Data Input Module (DATA): Supports the import of genomic and breed-related data, as well as geographic sampling information.

(2) Dimensionality Reduction Module (DimRed): Provides dimensionality reduction tools to aid in the visualization and interpretation of complex data sets.

(3) Phylogenetic tree construction module (PhyloTree): Constructs evolutionary trees to visualize genetic relationships among breeds.

(4) Population Structure Analysis Module (Structure): Reveals genetic structure and historical patterns of population stratification.

(5) Machine Learning Model Training Module (TrainModel): Supports multiple machine learning algorithms to train efficient breed classification models and generate molecular identity cards for individual breeds.

(6) Model Usage Module (PredNewind): Allows users to apply trained models for accurate breed identification.

The rPIMS package has been developed and is maintained by Dr. Yuhetian Zhao of the Institute of Animal Science, Chinese Academy of Agricultural Sciences. Should any queries or suggestions for improvement arise, please direct them to Dr. Zhao at zyuhetian@163.com.

Installing R and R dependencies

The rPIMS package was developed using the R programming language. Therefore, it is necessary to download and install R from the Comprehensive R Archive Network (CRAN) (www.r-project.org) before running mapDATAge. In order to optimise the user experience, it is recommended that RStudio Desktop is downloaded and installed, which is available at www.rstudio.com. The mapDATAge package is dependent on a number of R libraries and requires the R version 4.3.2 or above. Once the R environment is installed, the various dependencies can be installed using the following commands:

```
install.packages("BiocManager")

install.packages(c("shiny", "shinythemes", "shinyjs", "DT", "pwr", "dplyr", "FNN",
"shinyWidgets", "colourpicker", "RColorBrewer", "ggplot2", "data.table",
"sommer", "ape", "pbapply", "infotheo", "caret", "class", "randomForest", "xgboost",
"ranger", "ROCR", "smacof", "umap", "phangorn", "shinyalert", "leaflet", "e1071",
"kernlab"))

BiocManager::install("LEA")

BiocManager::install("ggtree")
```

Running rPIMS

We provide the rPIMS package on GitHub, available for download in the "Releases" section. You can find the rPIMS_0.1.0.tar.gz file (https://github.com/Werewolfzy/rPIMS/releases). This package is designed to streamline your data analysis and visualization tasks, offering a user-friendly interface for a range of functionalities.
To install the package locally, use the following command in R:

```
install.packages("path/to/rPIMS_0.1.0.tar.gz", repos = NULL, type = "source")
```

Once installed, simply run the package by executing:

```
rPIMS.GUI()
```

This command will launch the graphical interface, providing you with easy access to all the features and tools included in rPIMS. Enjoy exploring the capabilities of your new package!

Description of Example Files

To fully demonstrate the functionality and flexibility of rPIMS, we have prepared three example files, each with specific purposes and formats.
The first file is a genomic data file, sourced from this article (https://www.mdpi.com/2099408). To reduce computational load and processing time, we have limited the available dataset to individual genotypes from a randomly selected group of 10 breeds. This choice aims to provide a representative sample, allowing users to quickly engage with and experience the basic features of the software. During data processing, we carefully filtered out individuals and loci with missing genotypes to

enhance data integrity and processing efficiency. The numeric allele coding is based on the genotype data; for example, a sample with the "C/T" genotype at a specific locus is coded as "C" equals 0 and "T" equals 2, while "N" is coded as NA, and other alleles (like A and G) are coded as 1. The data has a .hmp.txt suffix. Users can convert .vcf files to .hmp.txt format for further analysis using the following command:

```
run_pipeline.pl -Xmx4g -vcf test.vcf -export test -exportType Hapmap
```

The second file is the breed information file. This file provides detailed breed classifications for each individual in the .hmp.txt file, ensuring that each individual has a clear and unique breed identifier. This information is crucial for subsequent analyses, as it allows users to compare and analyze data based on different breeds.
The third file is the location information file, which is optional. This file aims to provide specific geographic location information for each breed. Such location data assists users in contextualizing their analyses and visualizing data based on geographic distribution, enriching the overall analytical experience.

General Instructions

PANEL1: DATA
The DATA panel serves as the primary data input interface for the entire program, allowing users to seamlessly upload and manage their data files. Users can choose input files by clicking on buttons located on the left side, enabling the selection of Genotype files, Classification files, or an optional Location file. This flexible design ensures that users can tailor their data input according to their specific analytical needs.
Once a Genotype file is uploaded, the information is displayed on the right side of the panel, as illustrated in Document Figure 1. Users have the option to display either the head (the first 10 rows) or all rows of the Genotype data, providing a quick overview or detailed inspection as required. The format of the Genotype file is detailed in Table S1, which includes a header row with crucial columns such as rs#, alleles, chrom, pos, strand, assembly#, center, protLSID, assayLSID, panelLSID, and QCcode, along with individual IDs such as DM001, DM004, DM005, etc. This structured layout allows users to efficiently review the genetic information available in their datasets.

Table S1. Genotype file format overview

rs#	alleles	chrom	pos	strand	assembly#	center	protLSID
1:21717	A/G	1	21717	+	NA	NA	NA
1:23949	T/G	1	23949	+	NA	NA	NA
1:31587	C/T	1	31587	+	NA	NA	NA
1:41466	G/A	1	41466	+	NA	NA	NA
**assayLSID**	**panelLSID**	**QCcode**	**DM001**	**DM004**	**DM005**	**…**	**…**
NA	NA	NA	R	R	R	…	…
NA	NA	NA	T	T	T	…	…
NA	NA	NA	C	Y	Y	…	…
NA	NA	NA	G	R	G	…	…

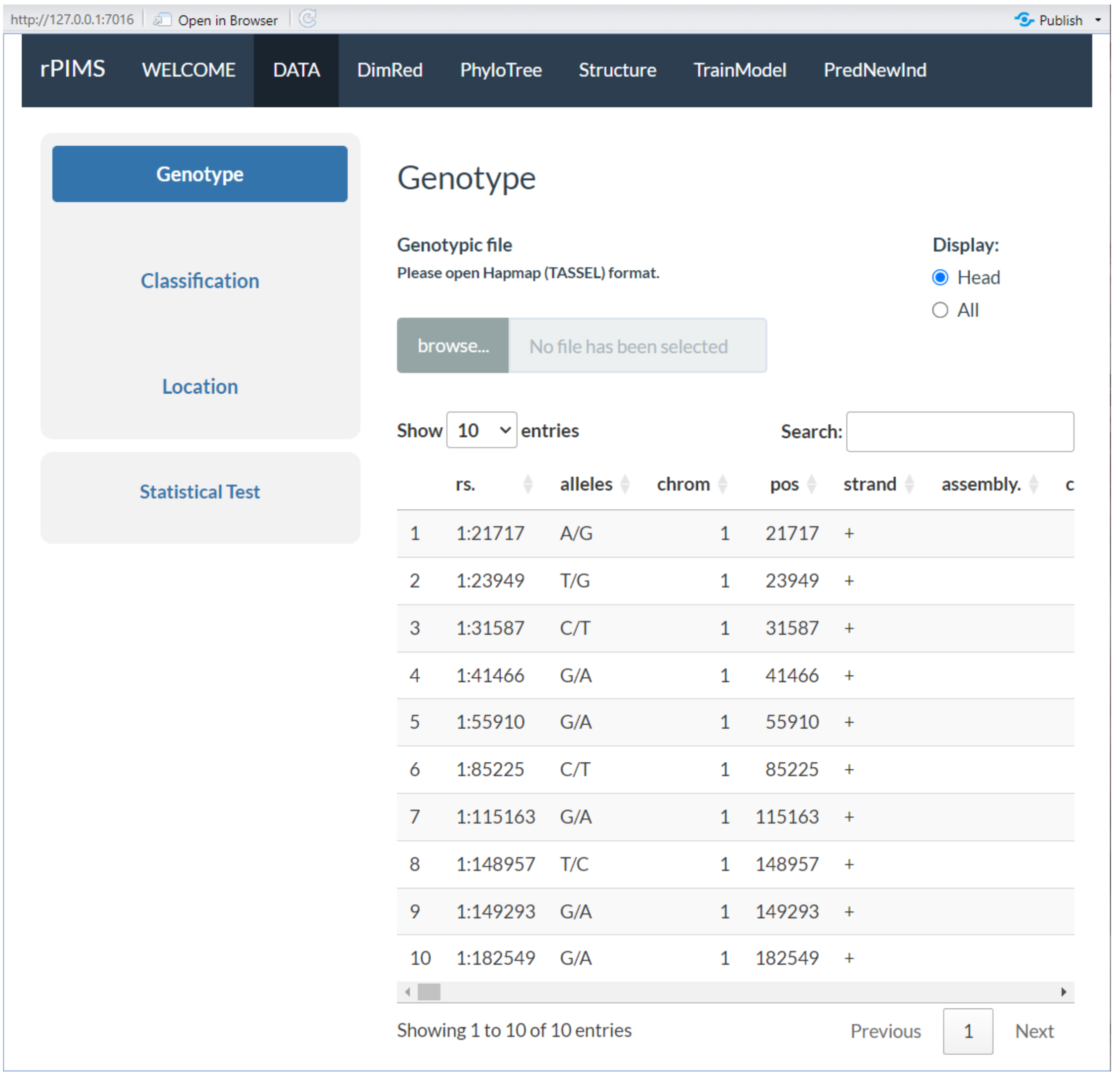

Document Figure 1. 'DATA' panel (Genotype).

Upon uploading a Classification file, the display on the right side updates to present its contents, as shown in Document Figure 2. Users can once again choose to view either the head or all data, allowing for flexibility in data examination. The format of the Classification file is specified in Table S2, featuring a header row that includes columns for ID and breed. This information is vital for ensuring that each individual genotype is associated with the correct breed classification, facilitating more accurate analyses.

Table S2. Classification file format overview

ID	breed
DM001	DormaSheep
DM004	DormaSheep
DM005	DormaSheep
DM006	DormaSheep
…	…

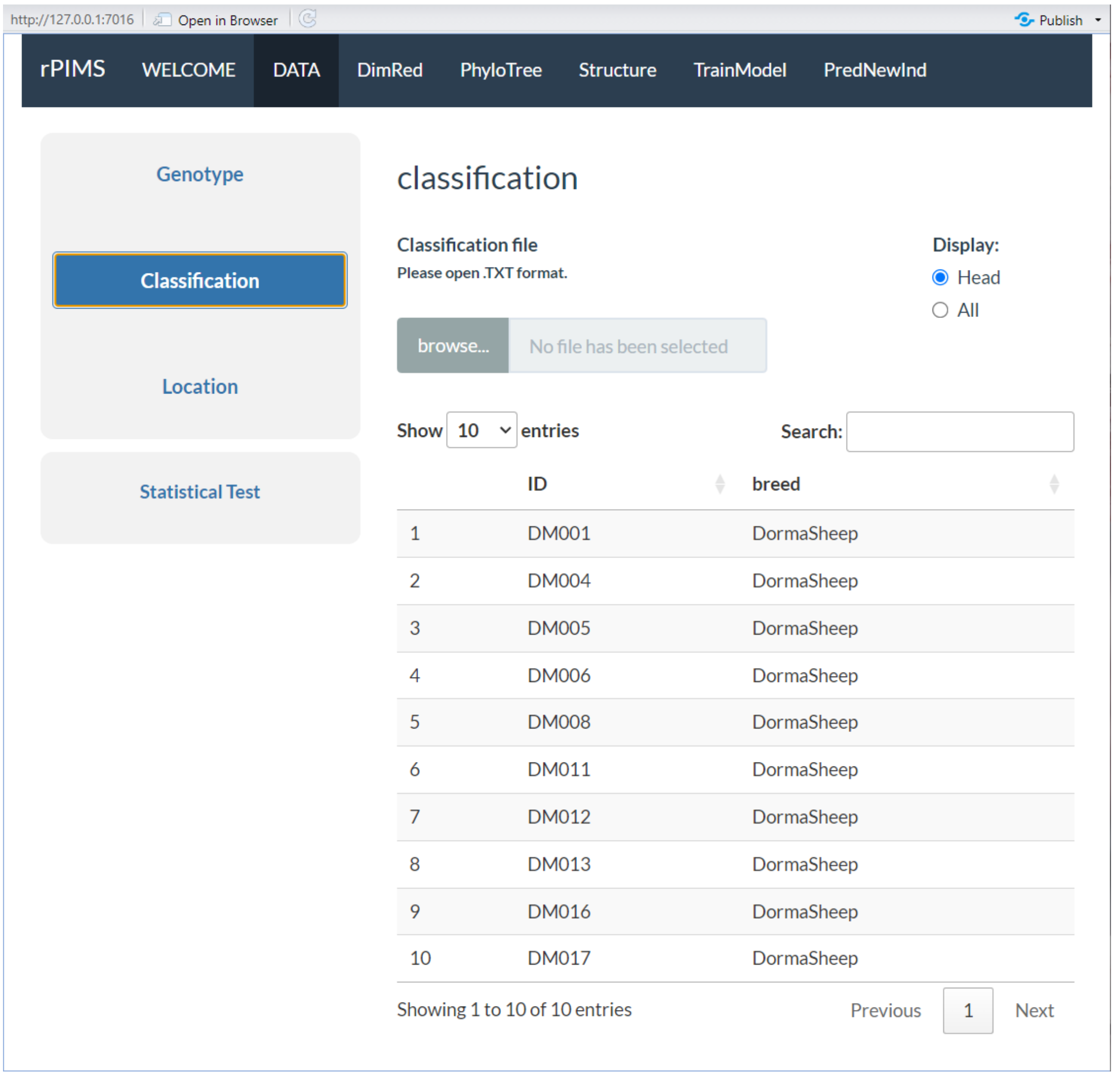

Document Figure 2. 'DATA' panel (Classification).

When a Location file is input, its contents are similarly displayed on the right side, as depicted in Document Figure 3. Users have the option to view either the head or all rows, enhancing their ability to quickly access relevant location information. The format for the Location file is outlined in Table S3, which includes a header row containing breed, Latitude, Longitude, and Location. This optional file enriches the dataset by providing geographic context, which can be critical for analyses that involve spatial considerations.

Table S3. Location file format overview

breed	Latitude	Longitude	Location
DormaSheep	32.2643	91.6843	Anduo County, Nagqu, Tibet
Gangbasheep	28.2756	88.5181	Gamba County, Shigatse, Tibet
Gonggasheep	29.9124	102.2332	Luding County, Garze, Sichuan
Huoerbasheep	29.7699	84.1589	Zhongba County, Shigatse, Tibet
…	…	…	…

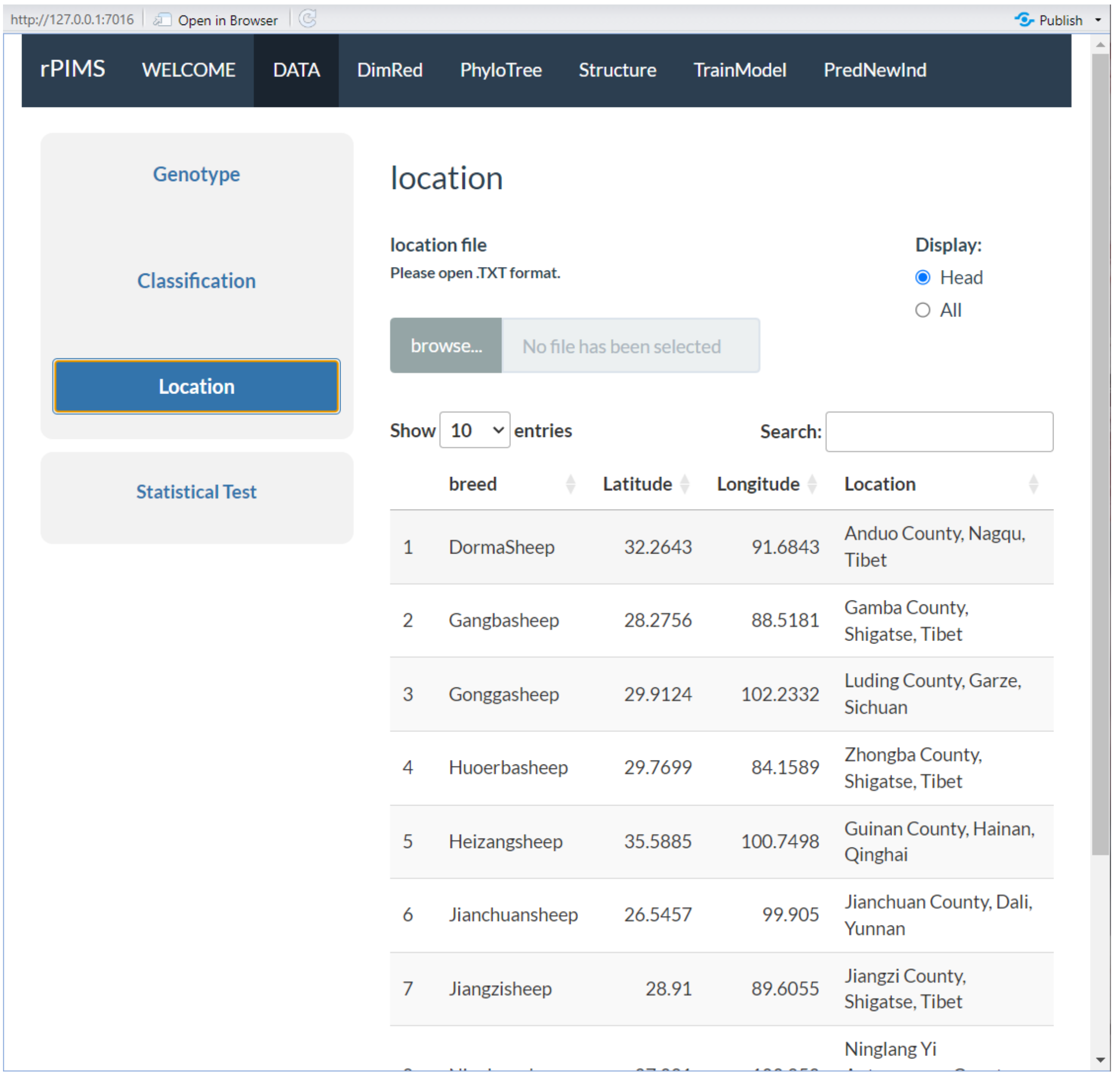

Document Figure 3. ‘DATA’ panel (Location).

After users input genomic data and breed-specific data, it is recommended that they perform statistical testing to validate the appropriate sample size required for each group, as shown in Figure S4. This enables users to confidently determine the number of samples needed for robust analyses. Users may customize the significance level (α) and statistical power parameters to generate tailored recommendations. Breeds with sample counts exceeding the recommended threshold will be highlighted in green, while those falling below the requirement will be flagged in red, providing immediate visual feedback on data adequacy.

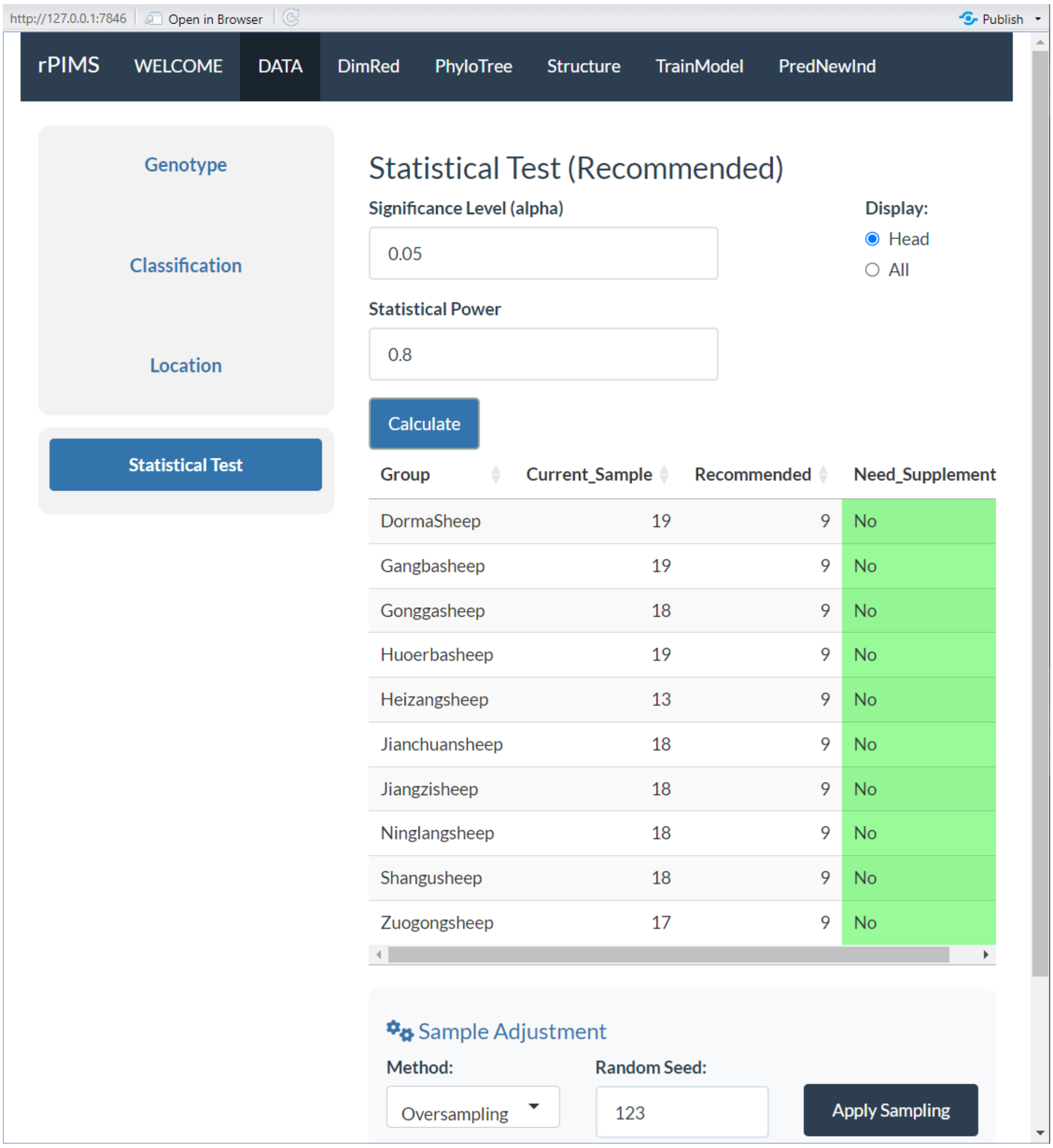

Document Figure 4. 'DATA' panel (Statistical Test).

Following statistical testing, our platform enables downsampling or upsampling (via duplication or SMOTE-based synthetic sample generation) to optimize group sizes, either aligning with Recommended Count (derived from user-defined significance level α and power) or matching the Group Average across breeds. Post-adjustment, groups meeting the threshold are highlighted in green, while undersized groups are flagged in red for immediate assessment (see Document Figure 5), ensuring analytical rigor and transparency in preprocessing.

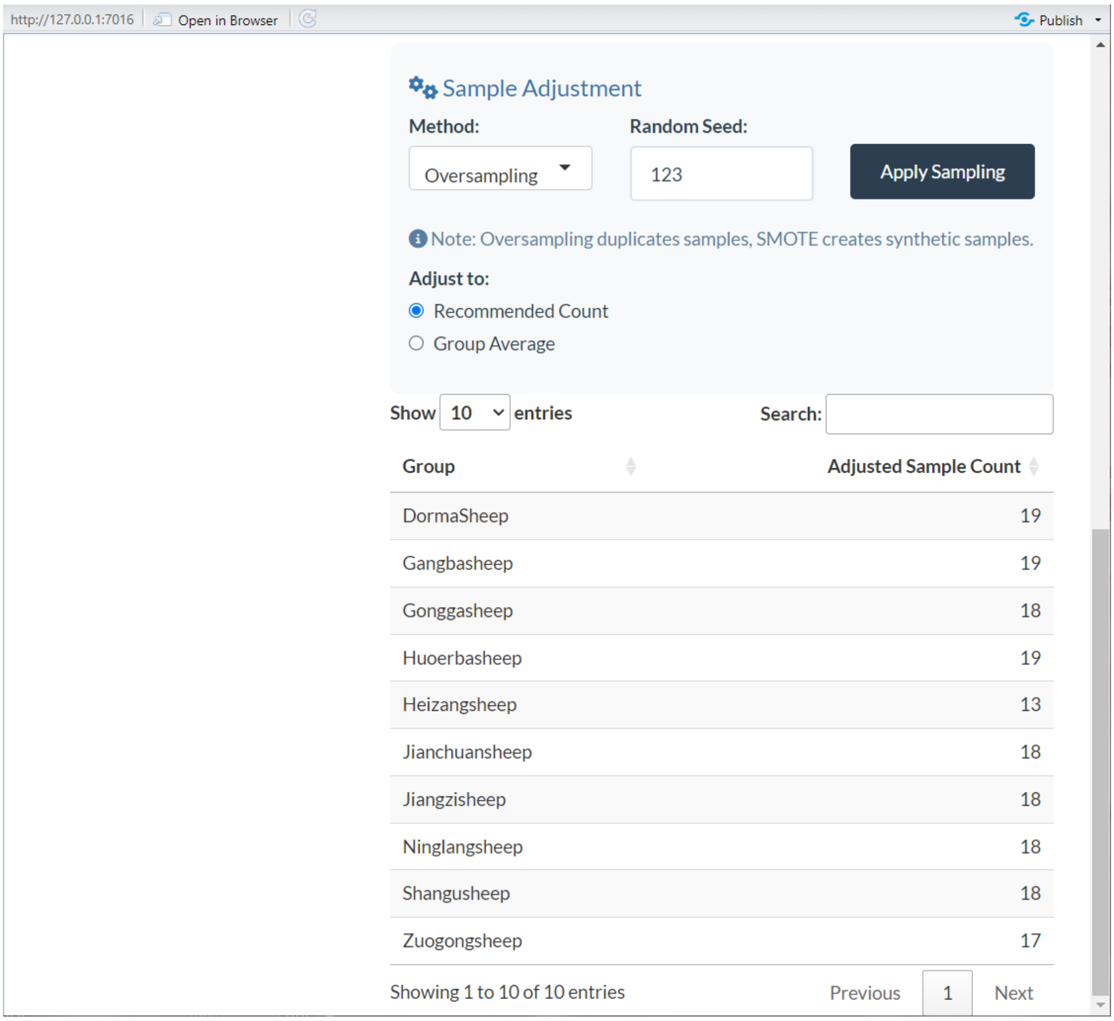

Document Figure 5. ‘DATA’ panel (Downsample or Upsample).

Overall, the DATA panel is designed to be user-friendly and efficient, enabling researchers to easily manage and explore their data, ensuring that they can conduct thorough analyses with confidence.

PANEL2: DimRed

The DimRed panel is designed to perform dimensionality reduction on genomic data, making it easy for users to visualize complex datasets in a simplified and intuitive way. This feature enables users to explore and interpret high-dimensional genetic data through various visualization options, enhancing their ability to identify patterns and relationships within the data.

1. Users can choose the desired dataset for analysis from a dropdown menu, as shown in label 1. Options include the Raw Data or other predefined datasets, allowing flexibility in selecting the input for dimensionality reduction.
2. Users can select a color scheme for the data points, as indicated in label 2. Multiple color modes are supported, such as RdYlBu, Spectral, etc., to effectively distinguish between different classification groups. This functionality helps in visualizing group-specific patterns and makes it easier to identify clusters or outliers within the data.
3. Users can select specific breeds for analysis using a multi-select option, as shown in label 3. This allows users to focus on particular breeds of interest and compare their genetic profiles within the reduced dimensional space. The multi-select functionality provides flexibility in including multiple breeds in the analysis simultaneously.
4. Users can choose from a variety of dimensionality reduction methods, including PCA (Principal Component Analysis) (Pearson, 1901), MDS (Multi-Dimensional Scaling) (Dixon, 2003), and UMAP (Uniform Manifold Approximation and Projection) (McInnes, et al., 2018), as indicated in label 4. Each method offers a unique perspective on the data, enabling users to explore different aspects of the genetic variation.
5. Users can select the dimensions to be displayed in the plot, such as PCA1_vs_PCA2 or PCA1_vs_PCA3, as shown in label 5. This feature allows users to observe data distribution across different principal components or other dimensions, providing insights into how different breeds or groups are related in the genomic space.
6. Users can choose the format for exporting the visualized data, including PDF, TIFF, PNG, JPEG, etc., as described in label 6. By clicking the “Download ZIP” button, all selected formats will be exported as a compressed file, making it easy to share and present the results.
7. The resulting plot displays the data distribution after dimensionality reduction based on the selected dimensions. As shown in label 7, data points representing different breeds will be distinguished according to the chosen color scheme, enabling users to clearly visualize the separation or overlap of different groups in the reduced space.
8. A table displays the dimensionality reduction results for all individuals, as indicated in label 8. Users can select different individuals from this table and click the “ReCalculate” button to update the visualization with the newly selected data. This interactive feature allows for dynamic exploration of the data and recalculation of the reduced dimensions based on user-defined subsets.

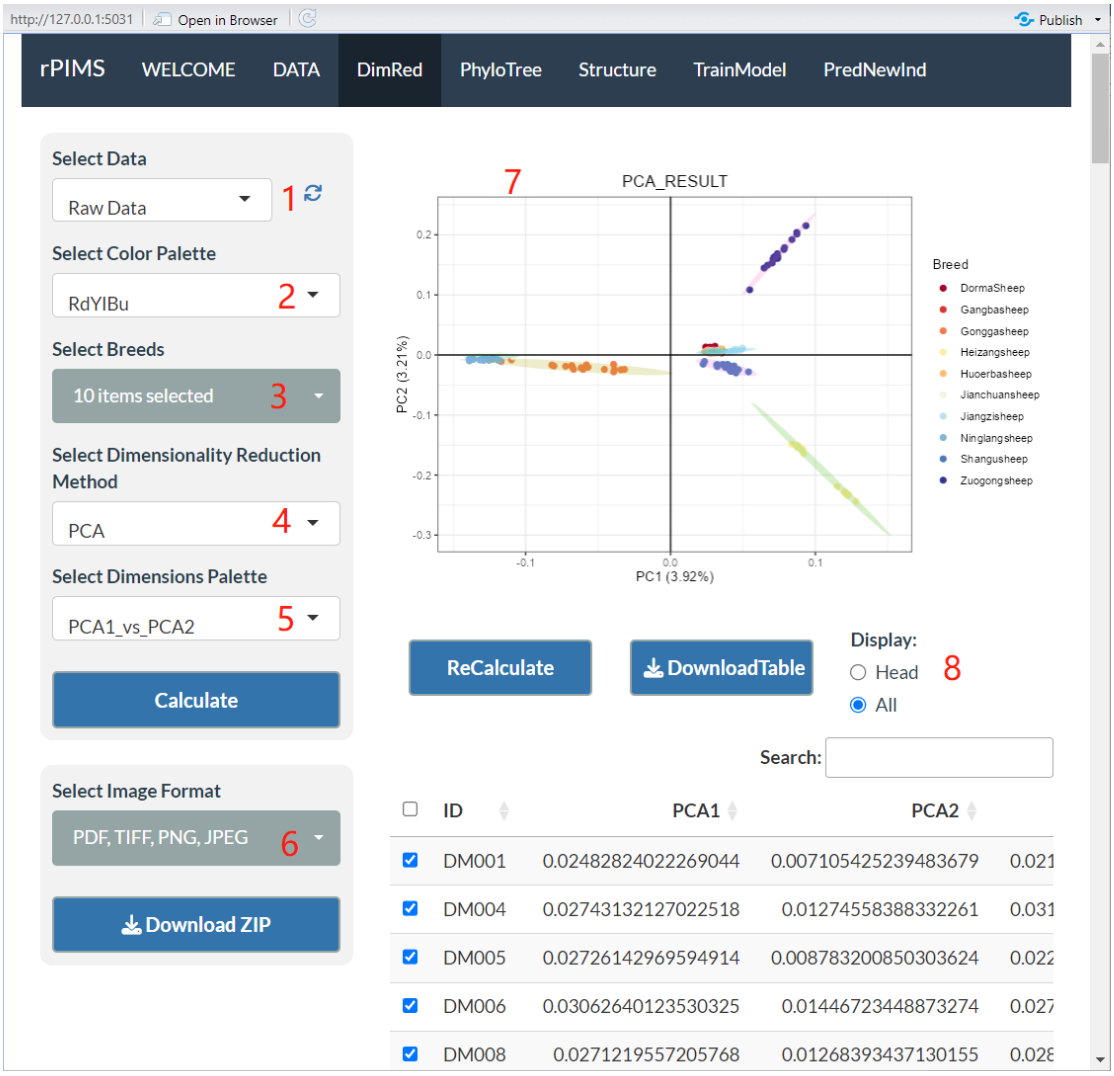

Document Figure 6. ‘DimRed’ panel.

PANEL3: PhyloTree

The PhyloTree panel is dedicated to conducting phylogenetic tree analyses on genomic data, providing users with an intuitive visual interface to explore evolutionary relationships. This panel enables users to construct and visualize phylogenetic trees with various customization options, making it easier to interpret and present the data.

1. Users can select the desired dataset for phylogenetic analysis from a dropdown menu, as shown in label 1. Options include Raw Data and other predefined datasets, allowing users to choose the most suitable input for constructing phylogenetic trees based on their research objectives.
2. Users can customize the color scheme for data points using various color modes such as RdYlBu and Spectral, as indicated in label 2. These color schemes help distinguish different classification groups within the phylogenetic tree, enhancing the visual clarity of the tree structure and making it easier to identify relationships among different groups.
3. Users can select multiple breeds for analysis using checkboxes, as shown in label 3. Selected breeds will be displayed in the generated phylogenetic tree, allowing users to focus on specific breeds of interest. This multi-selection capability enables a detailed comparative analysis of the evolutionary relationships among different breeds.
4. Users can choose from various phylogenetic tree (Paradis and Schliep, 2019) construction methods, such as Neighbor-Joining, FastME , BioNJ, and UPGMA, as indicated in label 4. Each method has unique advantages and is suited to different types of data, providing users with the flexibility to select the most appropriate method for their analysis needs.
5. Users can select the desired layout style for the phylogenetic tree, including options like rectangular_tree, slanted_tree, circular_tree, and radial_tree, as shown in label 5. Different styles offer various perspectives on the hierarchical structure of the tree and the relationships between breeds, allowing users to visualize the data in the most informative way.
6. Users can choose whether to perform Bootstrap analysis to evaluate the reliability of the phylogenetic tree, as indicated in label 6. Selecting "Yes" will compute Bootstrap values and display branch support rates on the tree, while selecting "No" will skip the Bootstrap calculation and generate a tree without support rates. This option is useful for assessing the statistical confidence of the phylogenetic relationships.
7. Users can select the format for exporting the phylogenetic tree, including PDF, TIFF, PNG, and JPEG, as shown in label 7. By clicking the “Download ZIP” button, all selected formats will be exported as a compressed file, allowing users to share and present their results conveniently.
8. The resulting phylogenetic tree graph displays the evolutionary relationships among different breeds, as shown in label 8. Users can customize the color scheme and tree style to better observe and analyze the tree structure. Each branch node's color represents a different breed, and the Bootstrap support rates indicate the reliability of each branch. This comprehensive visualization helps users to easily interpret the phylogenetic relationships.

9. A table displays the phylogenetic distances and branch lengths for all individuals, as indicated in label 9. Users can select different individuals from the table and click the “ReCalculate” button to update the phylogenetic tree with the newly selected data. This interactive feature allows for dynamic exploration of the phylogenetic relationships and recalculation of the tree structure based on user-defined subsets.

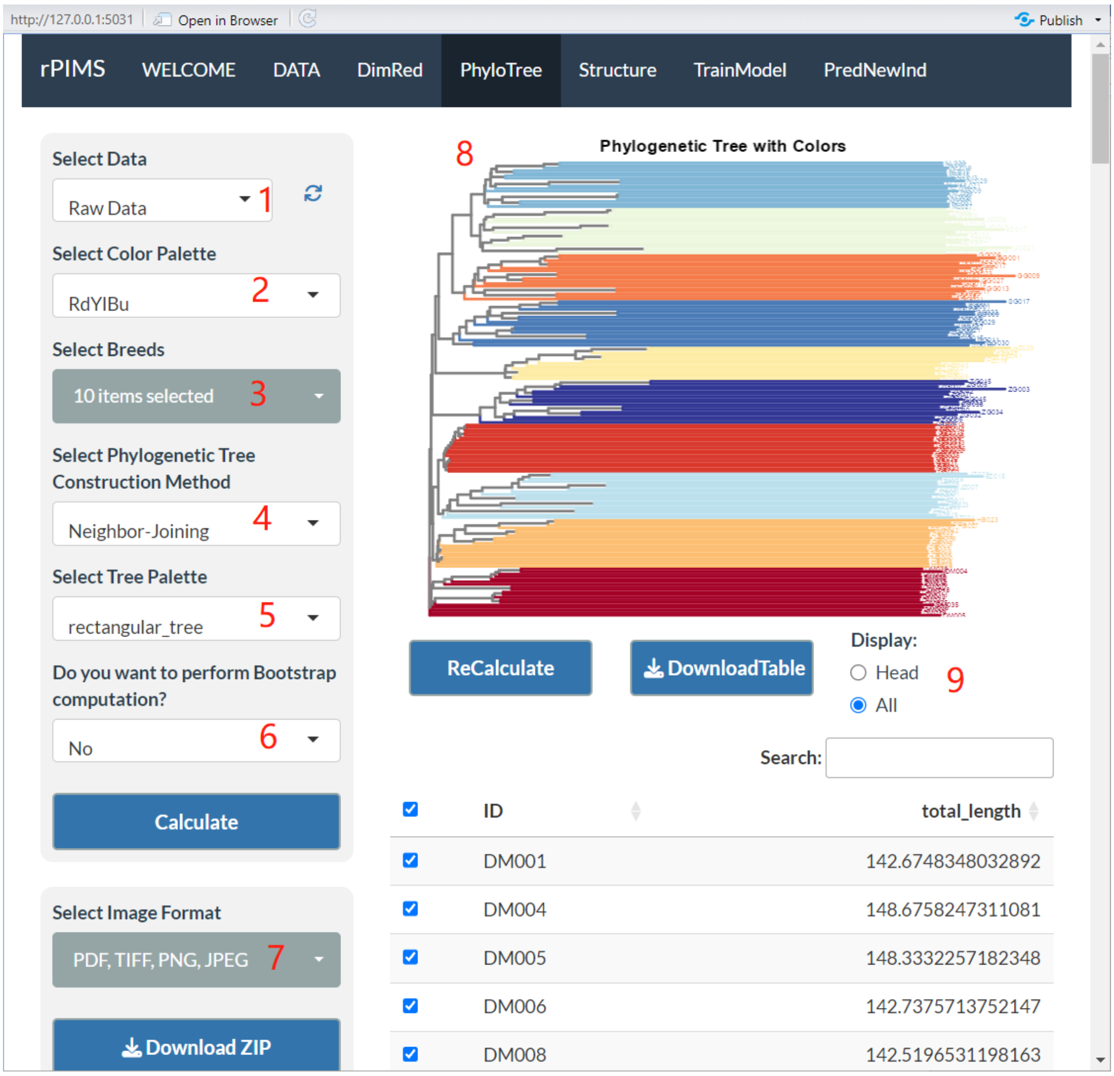

Document Figure 7. ‘PhylpTree’ panel.

PANEL4: Structure

The Structure panel is designed for performing population structure analysis on genomic data, providing users with a clear and user-friendly visualization of the genetic makeup of various populations. This panel enables users to easily investigate and interpret the ancestral components of individuals within a population, helping them to uncover genetic relationships and patterns.

1. Users can select the desired dataset for population structure analysis from a dropdown menu, as indicated in label 1. Options include the Raw Data or the data generated from previous analyses. This flexibility allows users to either work with unprocessed genomic data or utilize refined datasets for structure analysis.
2. Users can customize the color scheme for the structure plot, which represents different ancestral components, as indicated in label 2. Multiple color modes are supported, such as RdYlBu, Spectral, etc., to help distinguish between different classification groups or ancestral backgrounds. The choice of color scheme improves the visual clarity of the resulting structure plot, making it easier for users to differentiate between the genetic compositions of various groups.
3. Users can select multiple breeds for analysis by checking the corresponding boxes in the dropdown menu, as shown in label 3. The selected breeds will be displayed in the structure plot, allowing users to focus on specific populations of interest. This multi-select functionality makes it easy to compare the genetic structure of different breeds simultaneously.
4. Users can define the minimum and maximum K values for the analysis, as indicated in label 4. The K value represents the number of ancestral components, and it is a key parameter for population structure analysis. Users can set a range for K values to determine the optimal number of ancestral components by evaluating the cross-validation error.
4.1. Minimum K Value: This sets the smallest number of ancestral components for the analysis.
4.2. Maximum K Value: This sets the largest number of ancestral components for the analysis.
Cross-validation error helps users determine the most appropriate K value, representing the number of distinct ancestral populations present in the data.
5. Users can choose the format for exporting the resulting plots, including options such as PDF, TIFF, PNG, and JPEG, as indicated in label 5. Clicking the "Download ZIP" button will export both the cross-validation error plot and the structure plot as a compressed file, making it easy to share and present the analysis results.
6. The cross-validation error plot shows the error rates for different K values, as indicated in label 6. Users can analyze this plot to determine the optimal K value, which corresponds to the lowest cross-validation error. This optimal K value indicates the best estimate of the number of ancestral components, helping users refine their population structure analysis.
7. Once the optimal K value is selected, the population structure plot will display the

proportion of each individual's ancestral components, as shown in label 7. Different colors represent different ancestral components, and the X-axis represents the individuals from different breeds, while the Y-axis shows the proportion of each individual's ancestry. By analyzing this plot, users can gain insights into the genetic structure and ancestral relationships between different populations. The plot provides a clear and concise visual representation of how the genetic makeup varies across breeds.

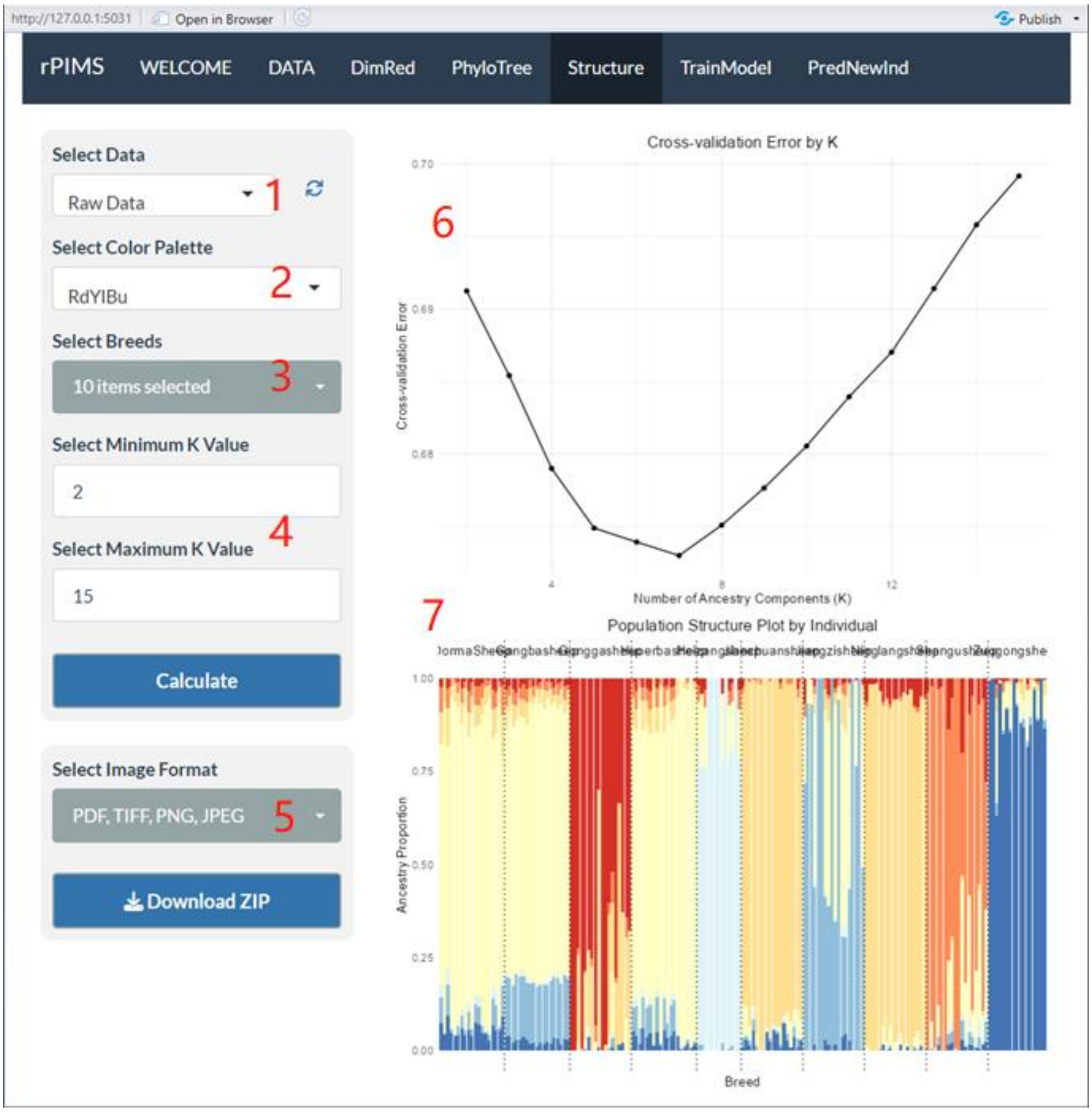

Document Figure 8. ‘Structure’ panel.

PANEL5: TrainModel

The TrainModel panel is the core component of the software, designed to build multi-breed classification models using significant SNP loci that show marked differences between breeds. The goal is to construct highly accurate classification models while minimizing the number of SNPs used. This section integrates statistical methods for differential analysis with a variety of machine learning techniques, enabling efficient selection of SNP loci for model building. These significant SNPs can also serve as valuable references for future biochip development. The panel is designed to be user-friendly, with a clear visual interface to guide users through the model training process.

1. Users can choose the dataset for model training and evaluation from a dropdown menu, as indicated in label 1. Options include Raw Data or datasets derived from previous analyses, allowing flexibility in selecting the appropriate input for classification model building.
2. Users can select a color scheme for the ROC curve to distinguish between different categories in terms of their ROC performance, as shown in label 2. Supported color modes include RdYlBu, Spectral, and others, providing visual clarity in evaluating model performance across categories.
3. Users can select multiple breeds for model training by checking the corresponding boxes in the dropdown menu, as indicated in label 3. This multi-selection feature allows users to train models on several breeds at once, enabling more comprehensive breed classification.
4. Users can set a random seed to ensure the reproducibility of results, as shown in label 4. By entering any integer value (e.g., 123), users can guarantee that the partitioning of the training and testing sets remains consistent across multiple runs.
5. Users can choose from several feature selection methods to filter out variables that have minimal impact on model prediction, as indicated in label 5. Available methods include:
5.1 Variance_Test (Kuhn, 2008): Filters variables based on their variance, retaining features with higher variance.
5.2 None: No feature selection is applied.
5.3 Chi-square_Test (R Core Team, 2013): Selects features based on their chi-square statistic.
5.4 F_Test (R Core Team, 2013): Uses the F-test to retain variables with significant group differences.
5.5 Mutual_Information_Method (Meyer, 2014): Selects features that have the highest mutual information with the target.
5.6 Fisher_Exact_Test (R Core Team, 2013): Applies Fisher's exact test to determine significant differences between categorical variables.
5.7 Pearson_Correlation_Test (R Core Team, 2013): Filters features based on their correlation with the target variable.
6. Users can specify the proportion of the dataset to be used for training and testing, as indicated in label 6. For example, a ratio of 0.8 means 80% of the data will be used for

training, and the remaining 20% will be used for testing.
7. Users can select from diverse cross-validation methods to validate model performance:
7.1 K-Fold Cross-Validation: The dataset is partitioned into K subsets, with each subset iteratively used as the validation set and the remaining K−1 subsets as the training set, ensuring robust evaluation of model generalizability.
7.2 Leave-One-Out Cross-Validation (LOOCV): A specialized case of K-fold where K equals the total sample size, leaving one sample as the validation set in each iteration, ideal for small datasets but computationally intensive.
7.3 Randomized Cross-Validation: The dataset is randomly split into training and validation sets multiple times, providing statistically robust performance estimates with reduced bias, particularly suited for large-scale datasets. 8. Users can set the number of cross-validation folds to evaluate the generalizability of the model, as indicated in label 8. Users can customize the number of folds (e.g., K in K-Fold) or iterations for cross-validation.
9. Users can choose from a variety of machine learning algorithms for model training, as shown in label 8. Options include:
9.1 KNN (K-Nearest Neighbors) (Venables and Ripley, 2013): A simple, instance-based learning algorithm used for classification.
9.2 Random_Forest (Liaw and Wiener, 2002): A powerful ensemble learning method that builds multiple decision trees for classification.
9.3 XGBoost (Chen, et al., 2016): An efficient and scalable implementation of gradient boosting for supervised learning tasks.
9.4 SVM (Support Vector Machines) (Meyer, et al., 2019): A robust algorithm used for classification by finding the optimal hyperplane that maximizes the margin between classes.
10. Users can select the format for exporting the results, including PDF, TIFF, PNG, and JPEG, as indicated in label 9. By clicking the "Download ZIP" button, all selected formats will be exported as a compressed file for further analysis and sharing.
11. Users can choose the format for exporting the trained model file, such as RDS, which allows easy storage and later reproduction of the model in the R, as shown in label 10.
12. The ROC curve helps users evaluate the classification model’s performance, as indicated in label 11. The X-axis represents the False Positive Rate (FPR), while the Y-axis represents the True Positive Rate (TPR). A curve that approaches the top-left corner indicates a well-performing model.
13. The confusion matrix provides a comparison between the model's predictions and the actual classifications, as shown in label 12. It shows the number of correct and incorrect predictions for each class, helping users assess the model's accuracy. Additionally, overall statistical information such as model accuracy, Kappa coefficient, and p-values are provided for a more in-depth evaluation of the model's performance.
14. Statistical performance metrics are provided for each breed-specific model:
14.1 Class: The breed category evaluated in the model, representing the target classification group.

14.2 Sensitivity (True Positive Rate): Proportion of true positive instances correctly identified, quantifying the model’s ability to detect target cases.
14.3 Specificity (True Negative Rate): Proportion of true negative instances accurately classified, reflecting the model’s capacity to exclude non-target cases.
14.4 Recall: Synonymous with sensitivity in binary classification, calculated as the ratio of correctly predicted positives to all actual positives.
14.5 Kappa (Cohen’s Kappa): Statistical measure of inter-rater agreement between predicted and actual classifications, adjusted for chance agreement.
14.6 Precision: Ratio of correctly predicted positive observations to the total predicted positives, indicating prediction reliability.
14.7 F1 Score: Harmonic mean of precision and recall, balancing both metrics to assess classifier performance under imbalanced class distributions.
15. After model training, the importance scores of each feature (SNP) are displayed, allowing users to identify which features contribute most to the model's predictions, as indicated in label 13. The higher the feature importance score, the more influence it has on classification decisions. Users can adjust a slider to view the top-ranked features, or they can input a specific number to display the top N important features. By clicking the “ReCalculate” button, users can retrain the model using only the selected features, aiming to minimize the SNP set while maintaining high accuracy.

The TrainModel panel combines advanced statistical techniques and machine learning algorithms to provide a robust platform for constructing and evaluating classification models. By offering flexible options for feature selection, cross-validation, and algorithm choice, the panel empowers users to build highly accurate and efficient models tailored to their specific datasets. The interactive visualization features further enhance the user experience, making it easy to interpret and refine models for breed classification.

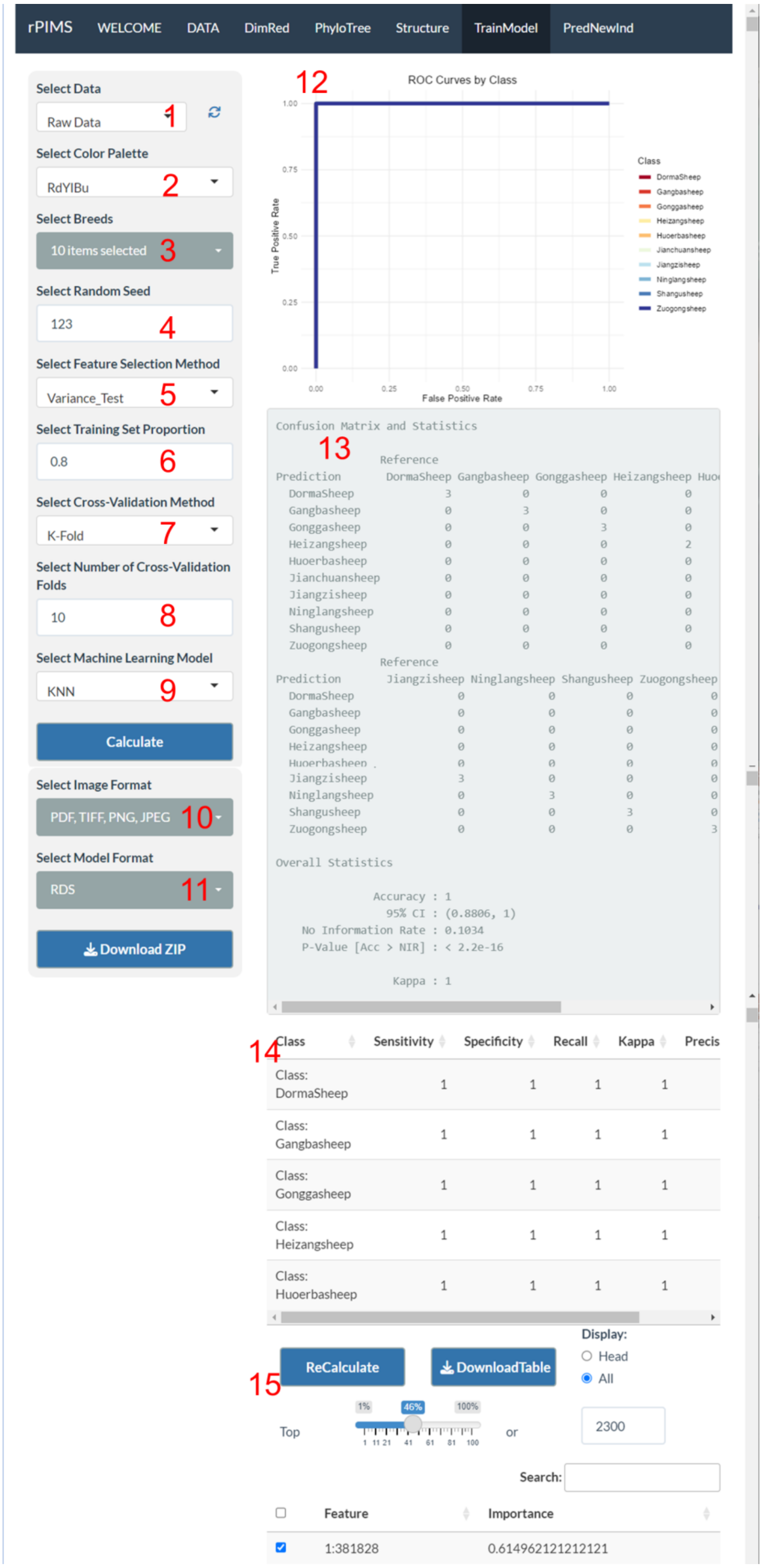

Document Figure 9. ‘TrainModel’ panel.

PANEL6: PredNewind

The PredNewind panel is the final part of the software, designed as an independent section that does not require computations from the previous panels. It allows users to accurately predict the breed information of unknown individuals based on genomic data, using a pre-trained model and SNP information. Additionally, this panel provides geographical distribution information of the predicted breeds and displays a map for easier interpretation. The panel is built with a user-friendly interface to streamline the prediction process.

1. Users can select a previously trained model file (such as an `.rds` file) by clicking the "Browse..." button, as shown in label 1. This model, constructed and saved from PANEL 5, can be reused without recalculating every time. Once the model file is selected, the filename will be displayed in this area, confirming that the model is ready for use in predictions.
2. Users can upload the genomic data file containing the SNP information of the individuals to be predicted by clicking the "Browse..." button, as indicated in label 2. The file format supports `.hmp.txt` and others consistent with the Table S1 format, which includes the genotype data for the SNP loci used in the model. The system will use this data to predict the breed of the unknown individuals.
3. The map displays geographical location markers based on the prediction results, as shown in label 3. Each blue pin represents the geographic coordinates of a predicted result. Users can zoom in and out on the map to examine the detailed geographic distribution of the predicted breeds. By clicking on a blue pin, users can view more detailed information about the predicted breed and its location. This visualization helps users to not only classify the unknown individuals but also understand their potential geographic origins.
4. A table displays the prediction results for each individual along with additional relevant information, as indicated in label 4. The table includes the following columns:
4.1 IID: The individual's ID.
4.2 predicted_result: The predicted breed for the individual.
4.3 z_score: A confidence score (ranging from 0 to 1), with scores closer to 1 indicating a higher prediction accuracy.
4.4 confidence: To enhance the reliability of predictions, we implemented a one-class validation framework to filter out predictions derived from random genotypes. The system provides dual designations: a "Reliable" classification indicates that the individual specimen resides within the model's validated predictive domain, while an "Uncertain" designation suggests potential extrapolation beyond the model's established prediction boundaries, thus precluding the generation of a z-score for such cases.
4.5 Latitude: The latitude coordinate of the predicted individual.
4.6 Longitude: The longitude coordinate of the predicted individual.
4.7 Location: A description of the specific location associated with the individual.

This table offers a clear overview of the prediction outcomes, allowing users to easily assess the classification results and associated confidence levels. The geographic

coordinates provide additional context for understanding the origins of the individuals.

In summary, the PredNewind panel is an essential tool for predicting the breed information of unknown individuals. By allowing users to input pre-trained models and genotype data, the panel enables accurate breed classification, while the interactive map and results table provide a clear and comprehensive understanding of the geographic distribution and confidence levels of the predictions.

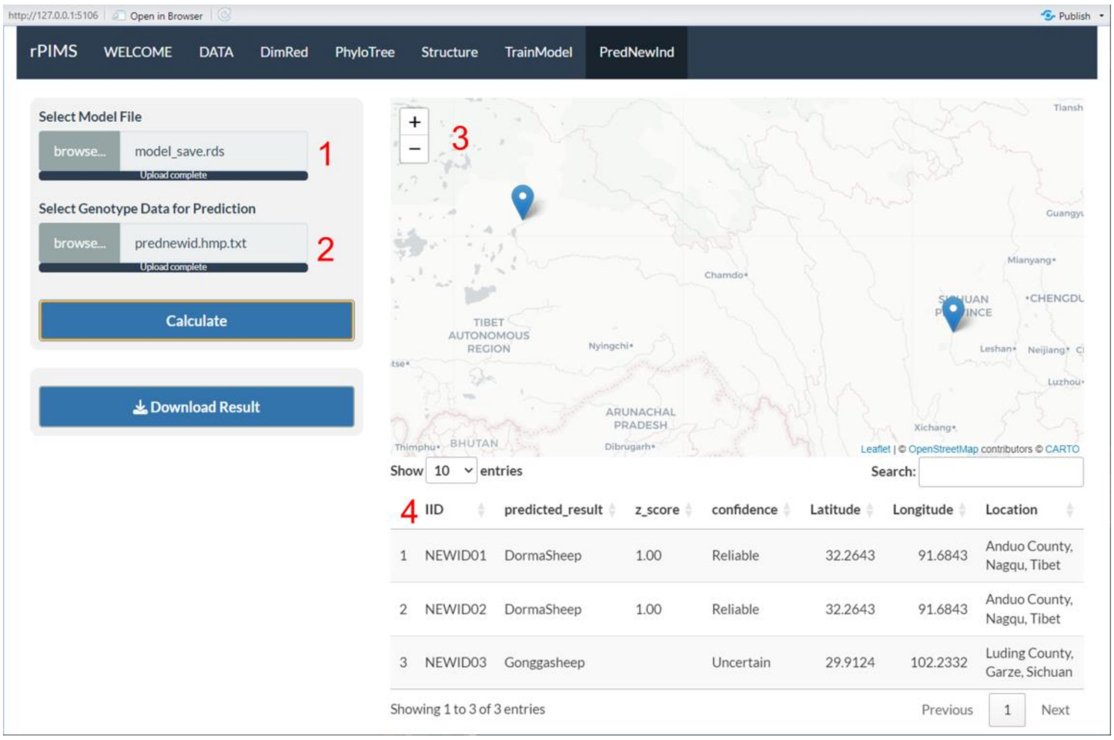

Document Figure 10. ‘PredNewind’ panel.

Chen, T., He, T. and Benesty, M. xgboost: Extreme Gradient Boosting. 2016.

Dixon, P. VEGAN, a package of R functions for community ecology. *Journal of vegetation science* 2003;14(6):927-930.

Kuhn, M. Building predictive models in R using the caret package. *Journal of statistical software* 2008;28:1-26.

Liaw, A. and Wiener, M. Classification and Regression by randomForest. *R News* 2002;23(23).

McInnes, L., Healy, J. and Melville, J. Umap: Uniform manifold approximation and projection for dimension reduction. *arXiv preprint arXiv:1802.03426* 2018.

Meyer, D., *et al.* e1071: misc functions of the department of statistics, probability theory group (formerly: E1071), TU Wien. *R package version* 2019;1(2).

Meyer, P.E. infotheo: Information-Theoretic Measures. Imagery, memory, and cognition :; 2014.

Paradis, E. and Schliep, K. ape 5.0: an environment for modern phylogenetics and evolutionary analyses in R. *Bioinformatics* 2019;35(3):526-528.

Pearson, K. LIII. On lines and planes of closest fit to systems of points in space. *The London, Edinburgh, and Dublin philosophical magazine and journal of science* 1901;2(11):559-572.

R Core Team, R. R: A language and environment for statistical computing. 2013.

Venables, W.N. and Ripley, B.D. Modern applied statistics with S-PLUS. Springer Science & Business Media; 2013.
